# Supplementary material for: Trends and Associations of Past-30-Day Cigar Smoking in the U.S. by Age, Race/Ethnicity, and Sex, NSDUH 2002–2020
Source: Int J Environ Res Public Health. 2023 Sep 6;20(18):6716. doi: 10.3390/ijerph20186716 (PMC10531240; doi:10.3390/ijerph20186716)
Supplement: Supplementary file 1 [file ijerph-20-06716-s001.zip › ijerph-2457420-supplementary.pdf]

**Supplementary Table S1**. Variable categorization and construction	
**Variable**	**Definition**
**Cigar smoking**[a]	
Past 30-day cigar smoking	Smoking a cigar on ≥1 day during the past 30 days (yes/no)
Age at first cigar use[b]	The age at which respondents first smoked part or all of any type of cigar
Frequency of cigar smoking[b]	The number of days during the past 30 days on which respondents smoked part or all of a cigar
**Sociodemographic variables**	
Sex	Male; female
Race/ethnicity	Non-Hispanic White; non-Hispanic Black; Hispanic non-Hispanic Other (i.e., Native American/Alaska Native, Native Hawaiian/Other Pacific Islander, Asian, more than 1 race)
Age	12-17; 18-20; 21-25; 26-34; 35+<br><br>12-20; 21+, for a subset of analyses
Income	<$20,000; $20,000-$49,000; $50,000-$74,999; $75,000+
Current school grade[c]	≤8th grade; 9th-12th grade
Educational attainment[d]	<High school diploma/GED; ≥high school/GED
Sexual identity[d]	Heterosexual; gay or lesbian; bisexual; don't know
**Other tobacco use variables**	
Past 30-day cigarette smoking	Smoking a cigarette on ≥1 day during the past 30 days (yes/no)
Menthol cigarette smoking[e]	Were cigarettes smoked during past 30 days menthol (yes/no)
Past 30-day nicotine vaping	Vaping nicotine or tobacco on ≥1 day during the past 30 days (yes/no)

Past 30-day smokeless tobacco use	Using smokeless tobacco products on ≥1 day during the past 30 days (yes/no)
**Other covariates**	
Health insurance coverage	Has some form of health insurance (yes/no)
Past-year illicit drug or alcohol abuse or dependence	Met criteria for past-year illicit drug or alcohol use disorder (yes/no)
General overall health quality	Good/very good/excellent; fair/poor
Lifetime service in U.S. armed forces[d]	Yes/no
Past-month serious psychological distress[d]	Scores >13 were categorized as being indicative of serious psychological distress in the past month (yes/no)

[a] The NSDUH questionnaire prefaces the items on cigar smoking with the following text: "By cigars we mean any kind, including big cigars, cigarillos, and even little cigars that look like cigarette"

[b] Assessed among past 30-day cigar users only

[c] Assessed among youth aged 12-17 years only

[d] Assessed among adults aged 18 and older only

[e] Assessed among past 30-day cigarette smokers only

Supplementary Table S2. Characteristics of past 30-day cigar smokers, by age group— National Survey on Drug Use and Health, United States, 2020 (n=1,169; Weighted n=10,383,560)							
		Age					χ^2 p-value
		Age 12-17 (n=57; Weighted n=176,960)	Age 18-20 (n=123; Weighted n=711,650)	Age 21-25 (n=300; Weighted n=1,285,100)	Age 26-34 (n=285; Weighted n=2,538,010)	Age 35+ (n=400; Weighted n=5,671,840)	
		% (95% Cl)	% (95% Cl)	% (95% Cl)	% (95% Cl)	% (95% Cl)	
**Past 30-day cigar smoking**		0.71 (0.51-0.99)	5.76 (4.20-7.85)	6.08 (5.21-7.08)	6.26 (5.04-7.76)	3.19 (2.74-3.70)	<0.001
**Number of days smoked cigars in the past 30 days**	1-5 days	66.99 (47.98-81.71)	67.63 (50.62-80.98)	56.71 (48.18-64.86)	64.48 (53.11-74.42)	54.08 (45.36-62.55)	0.265
	6-19 days	21.18 (10.95-36.99)	10.77 (5.38-20.40)	21.00 (14.05-30.17)	11.23 (6.92-17.72)	15.16 (11.09-20.40)	
	20+ days	11.83 (3.21-35.20)	21.60 (10.57-39.11)	22.29 (16.62-29.21)	24.29 (15.69-35.61)	30.76 (22.67-40.23)	
**Age of cigar initiation**	Median (SE)	14.06 (0.38)	15.59 (0.59)	16.91 (0.33)	17.77 (0.30)	22.18 (1.17)	
**Past 30-day cigarette smoking**		39.11 (20.81-61.08)	34.72 (20.95-51.62)	55.41 (47.58-62.98)	45.91 (37.13-54.96)	47.69 (39.04-56.49)	0.201
**Past 30-day menthol cigarette smoking**[a]		66.28 (34.82-87.86)	55.30 (30.63-77.62)	52.41 (40.10-64.43)	56.39 (43.60-68.38)	53.14 (38.10-67.63)	0.926
**Past 30-day nicotine e-cigarette use**		39.11 (23.99-56.66)	25.07 (15.95-37.12)	32.02 (23.34-42.16)	8.15 (5.18-12.59)	4.82 (2.69-8.49)	<0.001
**Past 30-day smokeless tobacco use**		8.74 (3.07-22.45)	16.42 (5.97-37.82)	8.03 (4.80-13.12)	6.40 (4.08-9.89)	5.15 (2.78-9.34)	0.535
**Sex**	Male	64.20 (20.74-54.31)	72.82 (57.32-84.24)	63.03 (56.04-69.51)	71.10 (63.74-77.50)	75.14 (68.86-80.52)	0.168
	Female	35.80 (20.74-54.31)	27.18 (15.76-42.68)	36.97 (30.49-43.96)	28.90 (22.50-36.26)	24.86 (19.48-31.14)	
**Race/Ethnicity**	Non-Hispanic White	71.06 (49.46-86.03)	55.74 (39.27-71.04)	60.59 (53.18-67.54)	52.68 (43.91-61.29)	60.42 (52.28-68.01)	0.254
	Non-Hispanic Black	16.33 (5.75-38.43)	30.44 (15.77-50.57)	20.46 (13.94-29.00)	28.07 (19.82-38.12)	25.68 (19.06-33.64)	
	Hispanic	7.29 (2.10-22.34)	6.27 (2.68-13.96)	10.66 (6.37-17.32)	14.37 (8.72-22.78)	9.62 (5.86-15.39)	
	Non-Hispanic Other	5.32 (1.97-13.57)	7.55 (2.84-18.58)	8.29 (4.80-13.93)	4.88 (2.10-10.93)	4.29 (2.30-7.86)	
**Sexual identity**[b]	Heterosexual	-	84.49 (74.83-90.89)	76.53 (69.25-82.52)	87.08 (80.27-91.77)	94.98 (91.95-96.91)	<0.001

	Gay or Lesbian	-	0.62 (0.09-4.39)	3.09 (0.95-9.55)	5.42 (2.54-11.17)	0.58 (0.15-2.15)	
	Bisexual	-	14.89 (8.56-24.64)	19.55 (13.87-26.83)	6.98 (3.65-12.96)	3.78 (2.16-6.54)	
	Don't know	-	0.00	0.83 (0.11-6.19)	0.53 (0.13-2.14)	0.66 (0.16-2.72)	
**Completed education[b]**	<HS diploma/GED	-	29.08 (13.83-51.15)	11.20 (6.87-17.75)	9.68 (4.78-18.61)	12.34 (7.06-20.67)	0.270
	≥HS diploma/GED	-	70.92 (48.85-86.17)	88.80 (82.25-93.13)	90.32 (81.39-95.22)	87.66 (79.33-92.94)	
**Current school grade[c]**	≤8th grade	12.59 (5.06-28.01)	-	-	-	-	-
	9th-12th grade	87.41 (71.99-94.94)	-	-	-	-	
**Family income**	$0-19,999	6.33 (1.34-25.24)	32.71 (20.95-47.14)	29.22 (22.08-37.57)	19.51 (13.11-28.02)	25.69 (18.39-34.67)	<0.001
	$20,000-49,999	19.80 (8.95-38.27)	35.50 (22.49-51.08)	37.92 (29.61-47.00)	32.58 (25.75-40.25)	26.49 (19.55-34.84)	
	$50,000-74,999	13.69 (4.24-36.22)	7.77 (3.96-14.67)	16.06 (9.95-24.89)	18.55 (12.05-27.46)	12.12 (7.54-18.94)	
	≥$75,000	60.17 (37.84-78.95)	24.02 (13.48-39.07)	16.80 (11.24-24.35)	29.36 (22.19-37.71)	35.69 (29.32-42.61)	
**Ever military service[b]**		-	0.00	2.34 (0.82-6.51)	3.41 (1.58-7.19)	20.70 (13.58-30.24)	0.003
**Overall health status**	Excellent/Very good/Good	94.74 (87.03-97.97)	95.47 (85.14-98.72)	90.73 (83.83-94.87)	87.04 (79.69-92.00)	75.74 (68.67-81.64)	<0.001
	Fair/Poor	5.26 (2.03-12.97)	4.53 (1.28-14.86)	9.27 (5.13-16.17)	12.96 (8.00-20.31)	24.26 (18.36-31.33)	
**Has health insurance**		92.88 (72.65-98.46)	90.72 (78.06-96.41)	84.28 (75.40-90.37)	68.19 (56.20-78.17)	86.33 (80.51-90.61)	0.019
**Past month SPD[b,d]**		-	17.70 (9.98-29.45)	26.84 (20.63-34.12)	11.35 (7.22-17.40)	12.27 (8.35-17.68)	0.006
**Past year illicit drug or alcohol dependence/abuse**		61.14 (38.45-79.85)	30.29 (18.96-44.66)	43.49 (35.35-51.98)	21.50 (16.57-27.41)	20.53 (13.67-29.64)	<0.001

[a]Among persons reporting past 30-day cigarette use

[b]Among adults only

[c]Among youth only

[d]SPD = serious psychological distress
